# Prevalence and Determinants of Glycemic Abnormalities in Cardiac Surgery Patients without a History of Diabetes: A Prospective Study

**DOI:** 10.3389/fendo.2015.00125

**Published:** 2015-08-11

**Authors:** Roma Y. Gianchandani, Sima Saberi, Preethi Patil, Richard L. Prager, Rodica Pop-Busui

**Affiliations:** ^1^Division of Metabolism, Endocrinology and Diabetes, Department of Internal Medicine, University of Michigan, Ann Arbor, MI, USA; ^2^Ann Arbor Endocrinology and Diabetes Associates, PC, Ypsilanti, MI, USA; ^3^Department of Cardiac Surgery, University of Michigan, Ann Arbor, MI, USA

**Keywords:** cardiovascular surgery, hyperglycemia, diabetes mellitus, prediabetes, A1c, insulin resistance

## Abstract

**Objective:**

To evaluate the prevalence and persistence of postoperative glycemic abnormalities in patients without a history of diabetes, undergoing cardiac surgery (CS).

**Methods:**

Ninety-two patients without diabetes with planned elective CS procedures at a tertiary institution were evaluated preoperatively and 3 months postoperatively for measures of glucose control including hemoglobin A1c, fasting plasma glucose, 2-h post oral glucose load, and insulin levels. Data from the hospital course were recorded.

**Results:**

Valid data were available from 61 participants at 3 months; 59% had prediabetes and 10% had diabetes preoperatively by one or more diagnostic criteria and continued to be dysglycemic at 3 months. Preoperative A1C was an independent predictor of postoperative hyperglycemia (*p* = 0.02). Insulin resistance and BMI rather than glycemic abnormalities before surgery were associated with a longer duration of the postoperative insulin infusion (*p* = 0.004, *p* = 0.048).

**Conclusion:**

Seventy percent of CS patients without known diabetes met criteria for diabetes or prediabetes preoperatively, and these abnormalities persisted after surgery.

## Introduction

The prevalence of diabetes mellitus (DM) and impaired glucose tolerance (IGT) is rising and approximately 79 million US adults have an undiagnosed glycemic abnormality (1). Dysglycemia is reported to be an independent risk factor for cardiovascular (CV) events, especially in patients with coronary artery disease (CAD) and/or undergoing CV procedures (2, 3). These include increased length of stay, wound infections, mortality, and major cardiac events. The American Diabetes Association (ADA) now has three criteria to identify dysglycemia, which include a fasting plasma glucose (FPG), an oral glucose tolerance test (OGTT), and more recently added A1c. The relationship between pre-surgical glucose abnormalities and postoperative hyperglycemia is currently unclear. Some studies have shown elevated A1c levels affect postprocedure outcomes in patients with and without diabetes, especially after cardiac surgery (CS) and post coronary revascularization. Higher rates of deep sternal wound infections were noted by Alserius in their non-diabetes group if A1c was >6% (4). Corpus in his coronary revascularization patients without diabetes found that an A1C of 6–7% had a significantly higher rate of major adverse cardiac events (33 vs. 22%, *p* = 0.04), target vessel revascularization (31 vs. 19%, *p* = 0.02), and CV mortality (4.6 vs. 0.5%, *p* = 0.03) compared with non-diabetes patients with hemoglobin A1c levels <6% (5). An abnormal A1c level may therefore have prognostic significance in prediabetes patients (5). Unfortunately, some of this data are collected before A1c cutoffs for diagnosing diabetes were established leading to an overlap in the diabetes and prediabetes groups by current criteria. Currently, there are no intervention studies evaluating postprocedure outcomes after improving glucose control prior to the procedure, and therefore, algorithms to diagnose glycemic abnormalities preoperatively, and assess their long-term follow-up are currently not established.

The objective of this study was to evaluate prevalence of preoperative glycemic abnormalities in CS patients without known diabetes using the three ADA criteria, determine the course of dysglycemia peri- and postoperatively, and evaluate factors associated with their persistence.

## Materials and Methods

Sixty-one patients from the original cohort of 92 consecutive preoperative patients with no known diabetes, undergoing coronary artery bypass grafting (CABG), valve replacement, thoracic aortic procedures, or a combined procedure followed up for this study at a tertiary institution (6). Patients with known diabetes, organ transplantation, using glucocorticoids, or tube feedings were excluded. The University of Michigan Institutional Review Board approved the study, and all participants signed a written consent.

Demographic data, anthropometric measurements, FPG, hemoglobin A1c (A1C), 75-g OGTT, and insulin levels were obtained in all participants at baseline and 3-month postsurgery.

Glucose tolerance classifications recommended by the ADA were used (7). Normal glucose tolerance was defined as FPG <100 mg/dl and a 2HPG <140 mg/dl; impaired fasting glucose (IFG) was defined as FPG 100–125 mg/dl, and diabetes (DM) as ≥126 mg/dl. 2HPG during a 75 g. OGTT of 140–199 mg/dl was defined as IGT, and ≥200 mg/dl as DM. A1C of ≥6.5% was diagnostic of diabetes, 5.7–6.4% as prediabetes. Abnormalities of one or more of the three criteria FPG, A1C, or OGTT were considered as dysglycemia. Insulin sensitivity was assessed by the homeostasis model of assessment-insulin resistance (HOMA-IR), calculated by fasting Glucose (mg/dl) × fasting insulin (μU/ml)/405.

During the inpatient stay, patients who developed stress hyperglycemia, defined by either two consecutive point of care blood glucose (BG) levels 1 h apart >140 mg/dl (8) or one BG >200 mg/dl, were started on an institutional insulin infusion protocol (9). Infusion rates were titrated according to this protocol and their duration recorded. Once BG levels reached below 140 mg/dl the infusion was weaned and, if there was no subcutaneous insulin requirement, postoperative hyperglycemia was considered resolved.

The primary outcome of this study was the prevalence of dysglycemia preoperatively and 3 months postoperatively in CS patients, according to ADA standards (7). Secondary outcomes were the relationship between preoperative glucose abnormality and insulin sensitivity with incidence of persistent postoperative dysglycemia as documented by A1C and OGTT. Finally, the relationship between preoperative dysglycemia and the duration of insulin infusion was also evaluated. Length of the insulin infusion was divided into three categories; <24 h drip duration, 24–48 h drip duration, and >48 h drip duration.

### Assays

Plasma glucose and A1C were measured as described (6). Insulin was measured by a double-antibody radioimmunoassay using ^125^I-Human insulin tracer (Linco Research, Inc., St Charles, MO, USA).

### Statistical analysis

Statistical analysis was performed using PASW 18 (IBM, Armonk, NY, USA). A two sided *p*-value of <0.05 was considered as statistically significant. We compared the baseline characteristics, BG levels, and insulin sensitivity with the use of either *T*-test or chi-square test. The concordance between A1Cs, FPGs, and 2-HRs at admission and at 3 months were calculated using weighted kappa. Preoperative predictors like HbA1c, waist and hip circumference, which were associated with postoperative dysglycemia were entered into a logistic regression model. Kruskal–Wallis test was used to determine the relationship between preoperative dysglycemia and insulin infusion.

## Results

From the original cohort of 92 patients, data are reported for 61 participants with valid baseline and follow-up data (Table 1). Thirty-one participants were excluded due to loss to follow-up, canceled surgery, withdrawal of consent, death, or lack of complete data. The excluded group had similar baseline characteristics as those included.

**Table 1:** **Demographic and glycemic laboratory features of patients with and without any abnormality by the three different diagnostic criteria – A1C, FPG, and 2HPG**.

	Preoperative				Postoperative			
	Normal all criteria	Abnormal			Normal all criteria	Abnormal		
		A1c	FPG	2HPG		A1c	FPG	2HPG
*N* (%)	18 (30)	31 (51)	26 (43)	12 (20)	12 (20)	39 (64)	19 (31)	17 (28)
Age (years)	55 ± 13*	66 ± 12*	65 ± 11	67 ± 10	58 ± 13	62 ± 13	64 ± 10	67 ± 14
Male (%)	50	61	69	58	75	59	79	47
BMI (kg/m^2^)	30 ± 7.0	29 ± 6.5	29 ± 5.8	29 ± 6.0	32 ± 8.0	28 ± 5.9	30 ± 5.4	28 ± 7.5
Hip circum (cm)	113 ± 19	112 ± 19	111 ± 16	113 ± 14	110 ± 23	103 ± 17	103 ± 18	105 ± 15
Waist circum (cm)	104 ± 21	100 ± 20	101 ± 21	102 ± 17	103 ± 29	95 ± 20	99 ± 19	93 ± 18
Systolic BP (mm Hg)	119 ± 20*	133 ± 18	131 ± 17	128 ± 14	121 ± 16	127 ± 23	133 ± 20	129 ± 26
Diastolic BP (mm Hg)	70 ± 11	75 ± 11	74 ± 12	72 ± 15	69 ± 10	73 ± 12	74 ± 11	76 ± 16
FH of diabetes (%)	33	48	54	50	17	49	63	47
Hemoglobin A1C % (mmol/mol)	5.3 (34) ± 0.3*	6.0 (42) ± 0.3*	5.9 (41) ± 0.5*	5.8 (40) ± 0.6	5.0 (31) ± 0.4*	6.2 (44) ± 0.4*	5.8 (40) ± 0.5	6.0 (42) ± 0.6
FPG (mg/dl)	88 ± 8*	102 ± 18*	109 ± 15*	107 ± 23*	89 ± 11*	96 ± 11	109 ± 6.5*	100 ± 9.7
2HPG (mg/dl)	91 ± 24*	118 ± 44	127 ± 48*	181 ± 21*	106 ± 21	128 ± 61	138 ± 62	197 ± 36*
Insulin (IU)	21 ± 15	27 ± 59	31 ± 65	50 ± 92	20 ± 10	21 ± 13	24 ± 8.3	26 ± 17
HOMA	4.7 ± 3.7	8.8 ± 26	10 ± 28	18 ± 41	4.5 ± 2.5	5.0 ± 3.3	6.6 ± 2.2	6.3 ± 4.2
Insulin drip in hours	61	68	54	33	50	62	58	54

*Indicates *p*-value is significant at 0.05.

Patients who met any of the three criteria for prediabetes (59%) or DM (10%) preoperatively continued to have prediabetes (56%) or DM (23%) postoperatively. On analyzing each of the diagnostic criteria separately, 55% of patients with an abnormal A1C stayed in the prediabetes range and 21% remained in the DM range postoperatively. About 22% of those with an abnormal 2HPG remained prediabetic and 44% had DM postoperatively. Similarly, 38% of those with an abnormal FPG remained in the prediabetes range postoperatively.

The dysglycemic group was older than the group without dysglycemia (66 vs. 55 years, *p* = 0.001). There was no significant difference between the two groups in regard to other baseline demographics. A1C, FPG, and 2HPG levels were significantly higher for those who had dysglycemia by one or more of the three criteria than those who had no abnormality (Table 2). The group classified as normal by all three criteria preoperatively had a significantly lower systolic blood pressure (119 ± 19 mm Hg) compared to the dysglycemic group (133 ± 17 mm Hg).

**Table 2:** **Differences between patients without any glycemic abnormality and with glycemic abnormality by any one of the three criteria – FPG, 2HPG, or A1C**.

	Preoperative			Postoperative		
	Normal	Abnormal (all)	*p*-Value	Normal	Abnormal (all)	*p*-Value
Number of patients	18	40		12	49	
HgbA1c % (mmol/mol)	5.3 (34)	5.9 (41)	<0.001	5.1 (32)	6.0 (42)	<0.001
2HPG (mg/dl)	90.61	122.0	0.001	105.5	128.5	0.03
FPG (mg/dl)	87.55	101.9	<0.001	89.4	97.7	0.02
Insulin	21.37	25.3	0.76	19.8	21.1	0.72
HOMA	4.70	7.9	0.56	4.5	5.2	0.47

After testing all three ADA criteria, Table 3 shows the postoperative persistence of each glycemic abnormality separately. We then evaluated the agreement of these tests (κ = 0.39, *p* < 0.001) and similarily found 2HPG to have the highest correlation (*r* = 0.73, *p* < 0.001) pre and postoperative. By the described multiple regression model, only preoperative A1C was an independent predictor of postoperative dysglycemia (β = 2.2, *p* = 0.02).

**Table 3:** **Preoperative and postoperative incidence of glycemic abnormality by each criteria separately**.

	By A1c		By FPG		By 2HPG	
	*N* (%)	Abnormal (IGT + DM) %	*N* (%)	Abnormal (IGT + DM) %	*N* (%)	Abnormal (IGT + DM) %
Before surgery	48	51 (48 and 3)	57	42 (39 and 3)	80	20 (15 and 5)
After surgery	37	63 (51 and 12)	69	31 (31 and 0)	73	27 (14 and 13)

*IGT, impaired glucose tolerance; DM, diabetes mellitus; 2HPG, 2 h post glucose load; FPG, fasting plasma glucose*.

All 61 patients met criteria for initiating an insulin infusion immediately after surgery. Mean preoperative insulin resistance and BMI were both higher in the groups that had a longer duration of insulin infusion (*p* = 0.004, *p* = 0.048). There is no significant association between preoperative A1C, FPG, 2HPG, and the length of the insulin infusion.

## Discussion

In this heterogeneous CS cohort, we found a higher prevalence of undiagnosed prediabetes by the above three ADA criteria (59% preoperatively and 57% postoperatively). A study of CABG patients by McGinn, also found higher rates of prediabetes, (57% prediabetes and 11% diabetes) (10). McGinn et al. used A1c as the sole criteria for diagnosing diabetes and as our group has previously shown A1C identifies the largest number of patients. Other studies using FPG and OGTT have identified larger proportions of undiagnosed DM rather than prediabetes (11, 12). These difference can be explained by our studies use of all three criteria and the deliberate choice of a heterogeneous CS cohort, including those undergoing valve, aortic, and CABG operations, while others have evaluated patients with CAD alone (11, 12). If CABG patients alone were evaluated in our study, the incidence of DM would probably have been much higher.

Previous studies have found that FPG and 2HPG were not equivalent in diagnosing dysglycemia (13, 14) and 2HPG was a better predictor of CV events and mortality (12). After testing all three ADA diagnostic criteria, we could evaluate the agreement of these tests and similarly found 2HPG to have the highest correlation (*r* = 0.39, *p* < 0.001) pre and postoperative. Together these data suggest that although obtaining a 2HPG after an OGTT is more cumbersome, it may have a predictive utility exceeding that of FPG or A1C (3, 15). Theoretically, it may be expected that an operative correction of a cardiac abnormality should increase physical and exercise capacity and improve or normalize dysglycemia postoperatively. Unfortunately, the findings reported here did not support this hypothesis, as glycemic abnormalities persisted in the subjects included in this study, and were even slightly increased 3 months postoperatively. This postoperative persistence may suggest either a more advanced impairment of glucose metabolism in this cohort, or a too short follow-up post surgery that was insufficient to restore normal glucose tolerance.

Among several baseline characteristics, in multivariable analysis, preoperative A1C was the only independent predictor of dysglycemia 3 months after surgery. This was similar to the post myocardial infarction data of Norhammer (12) and also in line with data reported by Sato et al., who found an association between A1c and BMI with intraoperative insulin resistance (16). Similarly, Cammu found that pre-anesthesia BG levels of >110 mg/dl predicted higher intraoperative insulin requirements (17). However, unlike our study, in Cammu’s study, the diagnosis of diabetes was made by history only, and as such, patients who may have had diabetes or prediabetes by other criteria could have been missed. In addition, several patients without a history of diabetes underwent an off-pump CABG. Since off-pump patients are not cooled to any degree and there is no glucose load in the cardioplegic solution, insulin requirements would not be comparable with patients presented in this study (17).

In our cohort, all patients, irrespective of whether they had DM, prediabetes or normoglycemia by one or more criteria, had BG excursions above 140 mg/dl right after surgery, requiring an insulin infusion as per our institutional cutoff. The variation in the frequency of insulin infusions used between studies is probably due to the different institutional cutoffs to initiate a drip [cut-off 140 mg/dl in our study and 120 mg/dl in Ref. (17, 18)]. Several of the CS studies described above evaluate insulin resistance and requirements during surgery. Unfortunately, numerous confounding factors exist at that point including: type of surgery, hypothermia, cardioplegic solution use, vasopressors, steroids, type of anesthetics, and other realities. In our experience, the immediate postoperative duration of the insulin infusion is the best predictor of recovery from surgical stress and insulin resistance. We therefore have used the duration of postoperative insulin infusion as a surrogate for recovery and found that, patients who had longer infusion durations had higher preoperative mean BMI, insulin levels, and HOMA-IR, suggestive of higher insulin resistance. An earlier study has similarly found that a higher BMI predicted larger perioperative insulin requirement (17). We found no association between preoperative A1C, FPG, or 2HPG and the duration of insulin infusion.

Strengths of this study are the combined use of all sensitive measures for the diagnosis of glucose abnormalities, consistent with the ADA criteria (7), and obtaining evaluations longitudinally, before and 3 months after surgery. To the best of our knowledge this was not done before, as other studies in various groups of hospitalized patients have used either a reported history of diabetes, A1C or FPG levels for the diagnosis of glucose abnormalities (14, 19). This study also evaluated a truly heterogeneous CS population, which includes aortic surgeries, valve repairs/replacements, and CABG procedures providing a broader perspective on dysglycemia prevalence and persistence in the CS population. The main limitations are the small study size and the short duration of follow-up. We also lost a large number of patients to follow-up, which could influence interpretation of the results.

## Conclusion

Undiagnosed abnormal glycemic states were highly prevalent, found in 70% of our heterogeneous group of CS patients and persisted 3 months after surgery. Several studies have associated dysglycemia with poor outcomes (20). This highlights the importance of dysglycemia screening and post-discharge glucose follow-up in these patients. Studies of larger size and longer duration are needed to assess the correlation of this dysglycemia to the future development of diabetes and its complications.

## Author Contributions

RG, SS, and RP-B designed the project. RG and SS wrote and edited manuscript. RP-B obtained funding, contributed to discussion, and revised/edited manuscript for critical content. PP performed statistical analysis and contributed to discussion. RP was involved in study design and manuscript review. All authors approved final version of the manuscript.

## Conflict of Interest Statement

The authors declare that the research was conducted in the absence of any commercial or financial relationships that could be construed as a potential conflict of interest.
